# A Fixed Combination of Palmitoylethanolamide and Melatonin (PEATONIDE) for the Management of Pain, Sleep, and Disability in Patients with Fibromyalgia: A Pilot Study

**DOI:** 10.3390/nu16162785

**Published:** 2024-08-21

**Authors:** Riccardo Terribili, Giulia Vallifuoco, Marco Bardelli, Bruno Frediani, Stefano Gentileschi

**Affiliations:** Rheumatology Department, Siena University Hospital, Viale Mario Bracci 16, 53100 Siena, Italy; riccardo.terribili@gmail.com (R.T.); giuliavallifuoco94@gmail.com (G.V.); fredianibruno60@gmail.com (B.F.); gentileschi.reumatologia@gmail.com (S.G.)

**Keywords:** fibromyalgia, palmitoylethanolamide, melatonin, nutraceuticals, pain, sleep, quality of life

## Abstract

Fibromyalgia is characterized by chronic widespread pain, fatigue, and sleep disturbances. Recent theories attribute fibromyalgia to central sensitization syndromes, suggesting altered nociceptive processing leads to hyperalgesia and allodynia. Standardized effective treatments are currently lacking. Palmitoylethanolamide and melatonin have shown pain-relieving effects in chronic pain conditions, including fibromyalgia, with excellent safety. Our open-label study assessed the impact of a daily combination of 1200 mg of palmitoylethanolamide and 0.2 mg of melatonin on pain, sleep, and quality of life in fibromyalgia patients. Between June 2023 and March 2024, 50 patients (2016 ACR criteria) were treated and evaluated at baseline, 1 month, 3 months, and 4 months (1 month discontinuation). The assessments included VAS for pain, ISI for insomnia, HAQ for health assessments, and a tender points evaluation. The patients, averaging 54.12 years old with a 3:1 female-to-male ratio, showed significant improvements in VAS, ISI, and HAQ scores relative to their own baselines and a reduction in tender points at 1 and 3 months, which was maintained at 4 months. No adverse events were reported. This study is the first to demonstrate the efficacy of a palmitoylethanolamide and melatonin combination as an adjunct therapy in fibromyalgia, highlighting its potential to reduce pain and improve sleep and quality of life.

## 1. Introduction

### 1.1. Fibromyalgia (FM)

FM is a clinical condition characterized by chronic widespread pain, fatigue, sleep disturbances, and cognitive impairment (in the absence of objective organ damage) typically associated with a plethora of concomitant disorders such as headaches, depression, anxiety, irritable bowel syndrome, endometriosis, hypothyroidism, and vulvodynia [1].

While FM has long been recognized, recent attention has been increasingly drawn to it due to its high prevalence (more than 2% worldwide [2,3]) and the significant disability burden experienced by affected patients in their everyday lives [4].

Several clinical criteria have been proposed and developed over the years, starting with addressing the presence/absence of specific areas of increased tenderness (tender points, TPs), as a distinctive feature of this disease [5], and culminating in the current adoption of the Widespread Pain Index (WPI) and the Symptom Severity Scale as standard diagnostic tools [6].

Currently, FM is included in the spectrum of central sensitivity syndromes, a group of conditions in which the source of pain (defined in this case as “nociplastic”) is attributable to a dysfunctional transmission and elaboration of painful stimuli by the central nervous system [7,8].

More specifically, although the exact etiopathology of FM is still far from being understood, various mechanisms are regarded to contribute to the onset of the conditions of hyperalgesia (the perception of an increased intensity of pain) and allodynia (the perception of normally nonpainful stimuli as painful), which are the cornerstone of its clinical presentation. In fact, the activation of mast cells [9] and microglia [10], increased oxidative stress [11], elevated levels of proinflammatory mediators [12], intense or prolonged psychological stress and the subsequent disruption of the Hypothalamic–Pituitary–Adrenal axis with the development of a state of cortisol resistance [13], and imbalance in the secretion and signaling of crucial neuromodulators [14] (mainly serotonin and dopamine) are all considered potentially responsible for the generation of the conditions of peripheral and central sensitization, affecting nociceptors and spinal and supraspinal levels, respectively.

In addition to the need for a careful differential diagnosis with respect to many other potential confounding pathologies (rheumatological/autoimmune or neurological conditions), the treatment of FM poses the greatest challenge in its management. Currently, there is still no universally acknowledged therapy for the treatment of FM, despite the existence of evidence supporting the effectiveness of certain drugs in improving symptom domains and quality of life. International guidelines favor a multimodal approach based on physical therapy and physical exercise as the first line of treatment [15]. Furthermore, patients with FM frequently report a certain degree of intolerance to different kinds of medications (muscle relaxants, opioids, non-steroidal anti-inflammatory drugs), significantly narrowing down therapeutic alternatives.

In this context, supplements and nutraceuticals could represent a useful tool as add-on therapy, especially due to their excellent safety profile.

### 1.2. Palmitoylethanolamide (PEA)

PEA is an endogenous fatty acid amide from the group of N-acetylethanolamides (which also includes the cannabinoid receptor ligand anandamide and the satiety agent oleoylethanolamide) which has displayed in vitro and in vivo analgesic, anti-inflammatory, and neuroprotective effects [16]. Although commonly referred to as an “endocannabinoid”, its direct interaction with cannabinoid (CB) receptors has not yet been demonstrated, while the greatest part of its functioning seems attributable to its binding to the transcription factor Peroxisome Proliferator-Activated Receptor alpha [16]. Indeed, this signaling pathway involves the trans-repression of proinflammatory transcription factors such as the Nuclear factor kappa-light-chain-enhancer of activated B cells, with the inhibition of the release of inflammatory cytokines (namely tumor necrosis factor alpha and interleukins 1β and 6) [16]. Additionally, the mechanism of PEA’s action has been described as Autacoid Local Injury Antagonism (outlined by the observation of a reduction in mast cell degranulation induced by a local injection of substance P in the ear pinna of developing rats, after its systemic administration) [17]. Interactions with the Transient Receptor Potential Vanilloid 1 (TRPV1) Receptors and the orphan G protein-coupled receptor (GPR)-55 are indeed believed to play a role in PEA’s global biologic effect [16].

In 2022, a randomized, double-blinded, placebo-controlled crossover trial on healthy volunteers highlighted that PEA exerts its effect by reducing peripheral and central sensitization and increasing pain modulation [18].

### 1.3. Melatonin

Melatonin is a pleiotropic hormone produced by the pineal gland and it is considered to exert a potential anti-nociceptive effect [19]. Most of all, it is known for its crucial role in the regulation of circadian biology, contributing to maintaining an appropriate duration and quality of sleep [20].

There is a well-recognized bidirectional relationship between pain and sleep. In fact, it is known that pain can disrupt sleep but also that short or disturbed sleep lowers the pain threshold and increases spontaneous pain [21]. Non-restorative sleep (NRS), indeed, is a highly prevalent feature in patients with FM, for whom a prospective study by Affleck et al. showed that a night of poorer sleep leads to more pain on the following day and that a more painful day is followed by a night of poorer sleep [22].

### 1.4. Clinical Utility

It is reasonable, then, to predict a potential therapeutic effect of both these compounds on chronic pain and, more specifically, FM.

In fact, a fair number of trials have investigated the efficacy and safety of PEA in different conditions of chronic pain [23,24], and a systematic review and meta-analysis from Lang-Illievich et al. reported a pooled effect favoring PEA over placebo or active comparators in the analgesic treatment of chronic pain, with negligible side effects [25].

A 2022 randomized, double-blinded, placebo-controlled crossover trial on healthy volunteers highlighted that PEA exerts its effect by reducing peripheral and central sensitization and increasing pain modulation [18].

Up to now, only two trials have investigated the efficacy of PEA in patients with FM as part of a combination therapy, both showing positive results [26,27].

On the other hand, two different systematic reviews (2020 and 2023) reported an improvement of different FM symptom domains after the administration of variable doses of melatonin supplementations [28,29].

### 1.5. Objective

We designed a prospective pilot study evaluating the effects of a fixed association between 1200 mg of hydrodispersible PEA and 0.2 mg of melatonin (PEATONIDE^®,^, produced by Pharmaluce Srl in the facilities of Erbozeta Group in the Republic of San Marino, San Marino, Italy) in addition to previous pharmacological treatment on the pain, sleep, and quality of life of a group of patients with FM.

## 2. Materials and Methods

We enrolled consecutive patients (aged between 18 and 80 years old) treated in the Rheumatology Clinic of Siena University Hospital (between June 2023 and March 2024) with a diagnosis of FM (according to the 2016 ACR criteria [30]) receiving a pharmacological therapy perceived as not satisfactory, who were started on a fixed combination of hydrodispersible PEA 1200 mg and 0.2 mg melatonin (PEATONIDE^®^) in the form of 1 orosoluble stick a day at bedtime for 3 months. Patients diagnosed with malignancy or under adjuvant cancer therapy were not considered for enrollment, because of the potential confounding factor of these conditions on their pain evaluation.

Patients were evaluated at baseline (T0), and then at 1 month (T1), 3 months (T2), and 4 months (T3). During each visit, they were administered the pain Visual Analogue Scale (VAS), Insomnia Severity Index (ISI), and Health Assessment Questionnaire (HAQ), and a trained rheumatologist assessed the positivity of tender points (through digital palpation, applying a force of 4 kg/cm^2^). PEATONIDE was discontinued at 3 months to determine a potential maintenance of the efficacy of the treatment over time.

### Statistical Analysis

Descriptive statistics were calculated for the different variables, reporting absolute and relative frequency measurements, mean and standard deviation, and/or median and interquartile range. Correlation was tested with Student’s *t* test, assuming *p* < 0.05 as the significance threshold. Tukey’s test was used for post-analysis comparisons.

## 3. Result

A total of 50 patients (38 females and 12 males) were enrolled in the study, with a mean ± SD age of 54.12 ± 13.3 years old (patients’ characteristics at baseline are detailed in Table 1). The most prevalent comorbidities were hypothyroidism (26%) and anxiety–depressive disorder (26%). Almost half of the patients (44%) had a form of FM secondary to a rheumatic/autoimmune disease.

**Figure 1:**
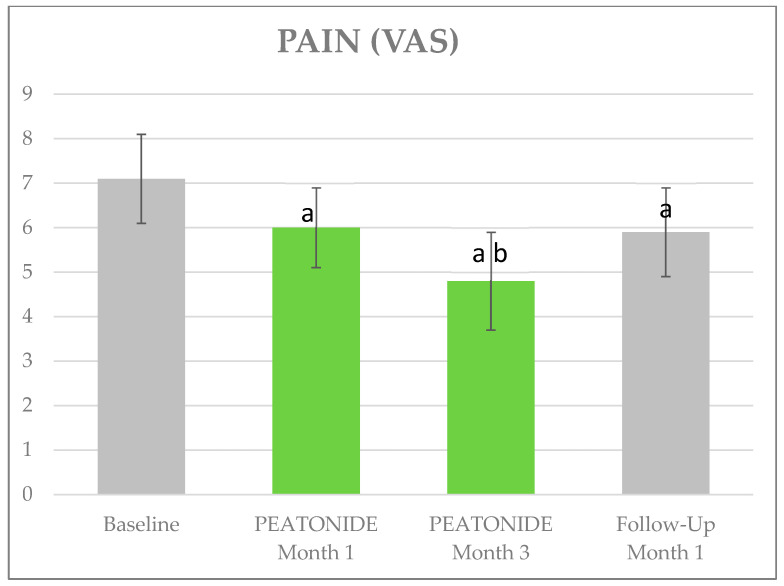
Effects of PEATONIDE on pain. a: *p* < 0.01 vs. baseline. b: *p* < 0.01 vs. PEATONIDE—Month 1.

**Figure 2:**
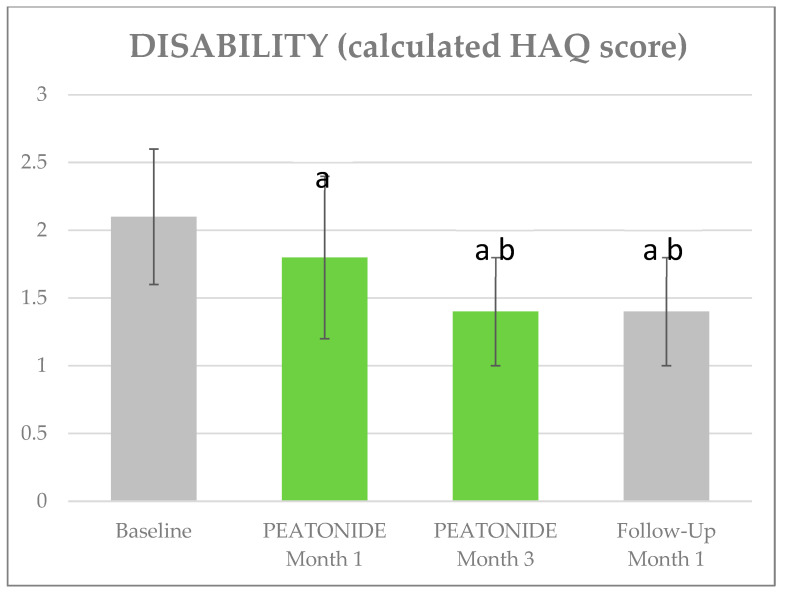
Effects of PEATONIDE on quality of life. a: *p* < 0.01 vs. baseline. b: *p* < 0.01 vs. PEATONIDE—Month 1.

**Figure 3:**
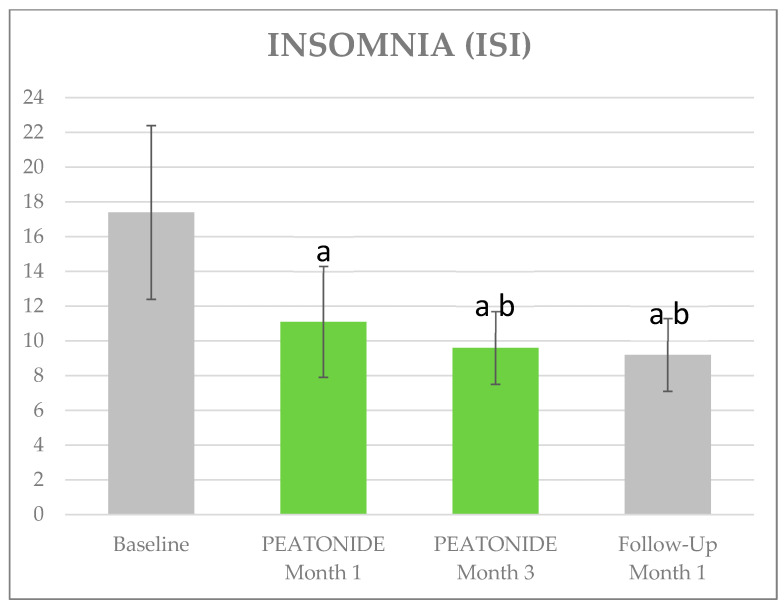
Effects of PEATONIDE on sleep. a: *p* < 0.01 vs. baseline and history. b: *p* < 0.01 vs. PEATONIDE—Month 1.

The VAS pain, ISI, and HAQ mean ±SD values at baseline were, respectively, 7.12 ± 0.96, 17.36 ± 4.95, and 2.11 ± 0.53; a mean of 15 TPs tested positive. Table 2 shows the trend in clinimetrics over the follow-up visits. At 1 month, the mean VAS pain improved by 1.16 points (5.96 ± 0.95, *p* < 0.01), at 3 months it improved by a further 1.18 points (4.78 ± 1.07, *p* < 0.01), and after 1 month of PEATONIDE withdrawal it worsened from the previous evaluation by 1.1 points (5.88 ± 1.02, *p* < 0.01). The ISI improved by 6.24 points at 1 month (11.12 ± 3.16, *p* < 0.01), by 1.54 points at 3 months (9.58 ± 2.13, *p* < 0.01), and finally by 0.36 points at 4 months (9.22 ± 2.05, *p* < 0.01). The HAQ improved by 0.295 points at 1 month (1.82 ± 0.55, *p* < 0.01), by 0.4 points at 3 months (1.42 ± 0.38, *p* < 0.01), and stabilized at 4 months (1.43 ± 0.37, *p* < 0.01). Finally, the average number of positive TPs decreased to 14 at 1 month, 13.5 at 3 months, and was 14 at 4 months (Figure 1, Figure 2 and Figure 3).

**Figure 4:**
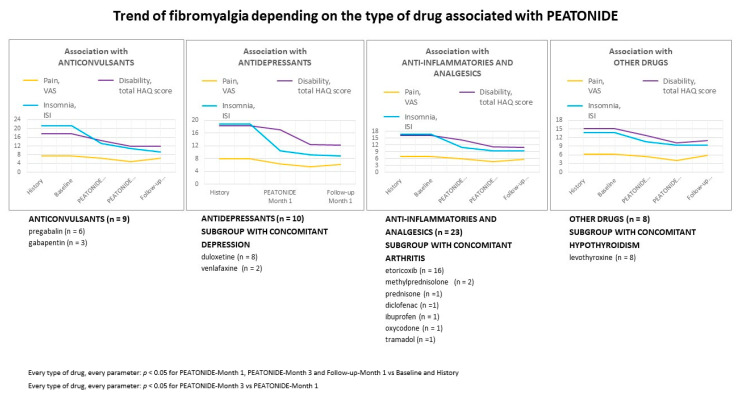
Trend in fibromyalgia depending on the type of drug associated with PEATONIDE.

The improvement of the different symptom domain scores was still present at 1 month after treatment suspension and did not appear to be influenced by the ongoing baseline treatment. In fact, the observed clinimetric variations remained consistent regardless of the type of drug associated with PEATONIDE, as demonstrated in Figure 4.

No adverse events were reported by patients or clinically observed during each follow-up visit.

## 4. Discussion

FM is a diffuse pathological condition characterized by an augmented perception of pain and other associated symptoms, especially sleep disturbances and daily fatigue. Recent etiopathogenetic theories address neuroinflammation as a major actor in the determination of central and peripheral sensitization in FM, leading to both hyperalgesia and allodynia. PEA and melatonin, two endogenous mediators, have been shown to exert analgesic and anti-inflammatory properties through various signaling pathways and have already been successfully used in the management of different chronic pain conditions and their associated symptoms [1,24].

Del Giorno et al. evaluated the effectiveness of the addition of ultra-micronized PEA to a previous Duloxetine (DLX) + Pregabalin (PGB) therapy in 35 FM patients, compared to the continuation of DLX + PGB therapy alone in other 45 patients, showing an improvement in VAS pain and TPs count at 6 months in the PEA arm [26]. According to a similar study protocol, Salaffi et al. reported better outcomes at 12 weeks in the WPI, Revised Fibromyalgia Impact Questionnaire (FIQR), and Fibromyalgia Assessment Status modified (FASmod) scores in FM patients started on PEA + acetyl-L-carnitine in addition to previous treatment with DLX + PGB, compared to patients continuing the previous treatment alone [27]. A recent review by Gonzalez-Flores et al. collected the results from six clinical trials assessing the use of melatonin alone or as an add-on therapy in FM and reported positive effects on TPs, pain intensity, sleep quality, fatigue, happiness, quality of life, and anxiety [29].

To our knowledge, this is the first study to explore the potential efficacy of a combination of melatonin and PEA on FM symptoms, and only the third study to evaluate PEA in FM. We observed a reduction in pain intensity (VAS) and an improvement in sleep quality and general quality of life after 1 and 3 months of treatment, with a maintenance of efficacy after 1 month of withdrawal, regardless of the baseline pharmacological therapy, which was continued alongside treatment with melatonin and PEA.

The dosage of melatonin in our study was lower than in previous trials, suggesting a synergistic effect with PEA. It is plausible that this synergy occurs at the level of mast cells, since on the one hand not only PEA but also melatonin can have inhibitory effects on mast cell activation [31], and, on the other, the activation of mast cells in the brain (in particular in the thalamus) has been described as an important physiopathological factor of FM [9]. Moreover, the combined action of melatonin on both sleep and pain, whose deep interplay has been highlighted in several rheumatic diseases and especially FM, could reasonably further explain its efficacy.

Of note is that, due to PEA’s high lipophilicity, micronized or ultra-micronized formulations are believed to be more easily absorbable, with more favorable pharmacokinetics and greater efficacy. Although there is some evidence supporting this theory, there is still no proof of the superiority of micronized PEA [16]. Our study could offer some more insight on this subject, since we made use of PEA that was not micronized but rather complexed with an amphipathic agent in order to make it water-dispersible and therefore more bioavailable, and it was seen to be effective as well.

In conclusion, the strengths of our study were mostly its originality (the combination of PEA and melatonin, PEATONIDE) and the enrollment of a quite large sample of patients. Weaknesses, on the other hand, might be found in the absence of a control group and the inclusion of patients with secondary FM, which could act as a possible confounder.

## 5. Conclusions

This was the first clinical study to explore the potential efficacy and tolerability of a combination of PEA and melatonin as add-on therapy in FM patients, showing a statistically significant lasting improvement in pain intensity, quality of sleep, and QoL, with no side effects. These results could strengthen the already existing body of evidence favoring the use of nutraceuticals in the management of chronic pain conditions and FM, for which it is often challenging to reach adequate disease control with standard therapies, offering an alternative to pharmacological polytherapy, which tends to be scarcely tolerated in these patients.

## Figures and Tables

**Table 1:** Baseline characteristics.

Patient No.	Sex	Age	Previous Treatment	Thyroid Dysfunction	Rheumatic/ Autoimmune Disease	Depressive–Anxiety Disorders	Other
1	F	34	Pregabalin	Hypothyroidism	-	-	-
2	F	56	Tramadol + dexketoprofen	-	-	-	Hypertension
3	F	51	Oxycodone + paracetamol	-	AR	-	-
4	M	72	Etoricoxib	-	ARPS	-	-
5	F	61	Duloxetine	-	-	Depression	-
6	M	41	Etoricoxib	-	ARPS	-	-
7	F	51	Gabapentin	Hypothyroidism	-	-	-
8	F	42	Levo-thyroxine	Hypothyroidism	-	-	-
9	F	64	Venlafaxine	-	-	Depression	-
10	F	71	Diclofenac	-	AR	-	-
11	M	38	Etoricoxib	-	ARPS	-	-
12	F	58	Pregabalin	-	-	Depression	-
13	F	33	Etoricoxib	-	AR	-	-
14	F	55	Duloxetine	Hypothyroidism	-	-	-
15	F	68	Methylprednisolone	-	AR	-	-
16	M	52	Methylprednisolone	-	AR	-	-
17	F	73	Ibuprofen	-	-	-	Hypertension
18	F	59	Gabapentin	Hypothyroidism	-	-	-
19	F	65	Venlafaxine	-	-	Depression	-
20	F	51	Etoricoxib	-	AR	-	-
21	M	46	Gabapentin	-	ARPS	-	-
22	M	77	Etoricoxib	-	AR	-	-
23	F	64	Levo-thyroxine	Hypothyroidism	-	-	-
24	F	31	Prednisone	-	AR	-	-
25	F	78	Pregabalin	Hypothyroidism	-	-	-
26	F	54	Duloxetine	-	-	Depression	-
27	F	34	Duloxetine	-	-	Depression	-
28	F	48	Duloxetine	-	-	Depression	-
29	F	61	Duloxetine	-	-	Depression	-
30	F	66	Etoricoxib	-	AR	-	-
31	M	28	Etoricoxib	-	ARPS	-	-
32	M	31	Etoricoxib	-	ARPS	-	-
33	F	38	Levo-thyroxine	Hypothyroidism	-	-	-
34	F	41	Levo-thyroxine	Hypothyroidism	-	-	-
35	F	65	Levo-thyroxine	Hypothyroidism	-	-	-
36	F	71	Levo-thyroxine	Hypothyroidism	-	-	-
37	F	56	Levo-thyroxine	Hypothyroidism	-	-	-
38	F	44	Levo-thyroxine	Hypothyroidism	-	-	-
39	M	57	Etoricoxib	-	ARPS	-	-
40	M	67	Etoricoxib	-	ARPS	-	-
41	M	56	Etoricoxib	-	AR	-	-
42	M	47	Etoricoxib	-	AR	-	-
43	F	65	Etoricoxib	-	AR	-	-
44	F	67	Etoricoxib	-	ARPS	-	-
45	F	38	Etoricoxib	-	ARPS	-	-
46	F	65	Pregabalin	-	-	Depression	-
47	F	56	Pregabalin	-	-	Depression	-
48	F	51	Pregabalin	-	-	Depression	-
49	F	52	Duloxetine	-	-	Depression	-
50	F	68	Duloxetine	-	-	Depression	-

Legend: AR (Rheumatoid Arthritis), ARPS (Psoriatic Arthritis).

**Table 2:** Clinimetrics trend at follow-up visits.

Patient No.	Baseline (T0)				1 Month (T1)				3 Months (T3)				4 Months (T4)			
	VAS	Tender Points	ISI	HAQ	VAS	Tender Points	ISI	HAQ	VAS	Tender Points	ISI	HAQ	VAS	Tender Points	ISI	HAQ
1	8	16/18	15	1.25	7	14/18	11	1.125	5	13/18	11	0.875	6	15/18	11	1.125
2	7	15/18	13	1.000	7	14/18	12	0.875	6	14/18	11	0.875	6	16/18	11	1.125
3	8	16/18	19	2.375	7	15/18	15	2.375	5	14/18	11	2.000	5	14/18	11	1.125
4	7	14/18	11	1.25	7	15/18	11	0.875	6	13/18	9	0.875	6	15/18	10	1.000
5	8	17/18	22	2.375	7	16/18	16	2.375	7	14/18	12	1.750	8	13/18	11	1.125
6	6	14/18	15	1.25	6	15/18	11	1.125	7	14/18	9	0.875	7	14/18	8	1.125
7	7	15/18	19	1.25	7	14/18	12	0.875	6	14/18	9	1.375	7	15/18	8	1.125
8	8	16/18	22	2.25	6	14/18	18	1.750	6	14/18	13	1.125	6	14/18	13	1.375
9	8	16/18	23	2.375	7	16/18	15	2.000	6	15/18	12	1.375	6	16/18	12	1.375
10	7	14/18	18	1.875	6	13/18	11	1.750	6	14/18	12	1.125	6	13/18	13	1.125
11	8	15/18	15	2.5	6	13/18	9	2.000	6	14/18	12	2.000	6	14/18	12	2.000
12	7	16/18	25	2.25	7	14/18	9	2.000	5	15/18	12	2.000	6	15/18	12	2.000
13	7	14/18	17	2.375	6	14/18	12	2.375	5	13/18	12	2.000	7	13/18	13	2.000
14	7	15/18	22	1.875	6	14/18	8	1.750	5	14/18	8	1.375	7	15/18	8	1.125
15	9	17/18	23	2.25	7	16/18	9	2.000	3	13/18	6	1.750	4	14/18	8	1.375
16	8	16/18	15	2.5	6	16/18	12	2.000	3	15/18	11	1.750	3	15/18	9	2.000
17	6	14/18	14	1.375	5	14/18	12	1.125	3	15/18	11	0.875	4	13/18	8	1.125
18	7	16/18	18	1.875	5	14/18	11	1.125	4	13/18	11	1.125	5	13/18	9	1.125
19	8	15/18	22	2.25	5	15/18	13	2.375	6	13/18	11	2.000	6	15/18	8	2.000
20	6	13/18	14	2.25	5	13/18	8	2.000	4	13/18	8	1.750	7	13/18	9	2.000
21	8	16/18	21	2.5	5	16/18	12	2.375	5	14/18	12	1.375	7	14/18	8	1.375
22	6	15/18	8	1.375	6	13/18	5	1.125	5	13/18	5	1.375	6	13/18	5	1.125
23	7	14/18	14	1.875	6	13/18	11	1.125	3	14/18	9	1.125	5	14/18	9	1.375
24	9	17/18	21	2.75	7	14/18	12	1.750	5	14/18	9	1.125	5	15/18	9	1.375
25	6	13/18	25	2.25	7	13/18	15	2.375	4	13/18	9	1.375	5	13/18	9	1.375
26	8	15/18	9	1.000	6	15/18	6	0.875	5	15/18	6	1.125	5	14/18	6	1.125
27	8	16/18	11	2.75	7	14/18	6	2.375	5	13/18	6	1.375	7	15/18	6	1.375
28	8	16/18	12	2.25	6	14/18	6	2.375	6	15/18	6	1.375	6	16/18	6	1.125
29	8	16/18	16	2.625	8	14/18	8	2.375	5	13/18	9	1.375	6	15/18	7	2.000
30	7	16/18	15	1.375	5	14/18	8	1.125	6	13/18	6	1.125	6	14/18	5	1.125
31	7	16/18	19	2.375	6	14/18	12	2.375	6	13/18	12	1.375	6	14/18	9	1.375
32	7	16/18	16	2.625	7	14/18	11	2.375	5	13/18	11	1.125	6	15/18	11	1.375
33	6	14/18	12	1.75	6	14/18	8	1.125	4	14/18	8	1.125	6	13/18	8	1.125
34	6	14/18	11	2.375	6	14/18	8	1.750	3	13/18	8	1.125	5	13/18	8	1.375
35	7	14/18	19	2.25	5	14/18	11	2.375	5	13/18	8	1.750	6	14/18	8	2.000
36	6	15/18	15	2.000	5	14/18	13	1.750	4	15/18	12	1.125	7	16/18	12	1.125
37	5	15/18	8	1.375	5	14/18	8	1.125	4	13/18	8	1.375	6	14/18	8	1.375
38	5	15/18	9	2.25	4	15/18	7	1.750	4	15/18	8	1.375	6	15/18	8	1.125
39	7	15/18	12	2.625	5	14/18	8	2.375	5	13/18	6	2.000	7	14/18	8	2.000
40	6	14/18	14	1.75	4	14/18	11	0.625	4	13/18	9	0.875	5	14/18	9	1.125
41	6	13/18	18	1.75	4	13/18	11	1.750	4	13/18	9	1.125	4	15/18	9	1.125
42	6	14/18	20	2.000	5	14/18	11	2.000	4	15/18	9	1.375	4	14/18	9	1.125
43	7	15/18	21	2.375	6	14/18	14	2.375	4	13/18	9	1.375	6	15/18	9	1.125
44	6	13/18	23	2.625	5	13/18	15	2.375	3	13/18	12	2.000	7	14/18	12	2.000
45	7	15/18	18	2.375	5	15/18	8	2.000	3	13/18	7	1.375	6	14/18	9	1.125
46	8	17/18	22	3.125	6	14/18	15	2.000	5	13/18	11	2.000	7	14/18	8	2.000
47	8	16/18	26	2.625	7	14/18	18	2.375	5	14/18	11	2.000	7	15/18	9	2.000
48	8	16/18	25	2.625	7	14/18	16	2.000	5	14/18	11	1.125	7	13/18	9	1.125
49	8	16/18	24	3.125	6	15/18	12	2.375	4	14/18	11	2.000	5	16/18	12	2.000
50	8	16/18	22	2.25	6	15/18	15	2.375	5	13/18	11	1.750	5	15/18	12	2.000

## Data Availability

The data presented in this study are available on request from the corresponding author due to privacy reasons.
